# Fractional calculus in mathematical oncology

**DOI:** 10.1038/s41598-023-37196-9

**Published:** 2023-06-21

**Authors:** Tudor Alinei-Poiana, Eva-H. Dulf, Levente Kovacs

**Affiliations:** 1 Department of Automation, Faculty of Automation and Computer Science, Technical University of Cluj-Napoca, Memorandumului Str. 28, 400014 Cluj-Napoca, Romania; 2 University of Agricultural Sciences and Veterinary Medicine Cluj-Napoca, 3-5 Manastur Str., 400374 Cluj-Napoca, Romania; 3 Physiological Controls Research Center, Óbuda University, Budapest, 1034 Hungary

**Keywords:** Predictive medicine, Cancer models

## Abstract

Even though, nowadays, cancer is one of the leading causes of death, too little is known about the behavior of this disease due to its unpredictability from one patient to another. Classical mathematical models of tumor growth have shaped our understanding of cancer and have broad practical implications for treatment scheduling and dosage. However, improvements are still necessary on these models. The primary objective of the present research is to prove the efficiency of fractional order calculus in mathematical oncology, more specifically in tumor growth modeling. For this, a generalization of the four most used differential equation models in tumor volume measurements fitting is realized, using the corresponding fractional order equivalent. Are established the fractional order Exponential, Logistic, Gompertz, General Bertalanffy-Pütter and Classical Bertalanffy-Pütter models for a treated and untreated dataset. The obtained results are compared by Mean Squared Error (MSE) with the integer order correspondent of each model. The results prove the superiority of the fractional order models. The MSE of fractional order models are reduced at least at half in comparison with the MSE of the integer order equivalent. It is demonstrated in this way that fractional order deterministic models can offer a good starting point in finding a proper mathematical model for tumor evolution prediction. Fractional calculus is a suitable method in this case due to its memory property, aspect that particularly characterizes biological processes.

## Introduction

Cancer is one of the world’s deadliest diseases. According to the World Health Organization, there were an estimated 19.3 million new cases of cancer and almost 9.9 million deaths from cancer worldwide in 2020^1^. A lot of research is being done to find proper models and to apply modern control algorithms to discover personalized treatment for patients to, at least, postpone cancer’s evolution with minimal impact of treatment side effects (chemotherapy, radiation therapy etc.).

Mathematical modeling is a powerful tool used in finding different structures that can predict the behavior of a system, even when referring to a living organism^2,3^. Using tumor mathematical models, it can be useful in cancer proliferation and survival signaling, tumor immunology, tumor microenvironment, metastasis and anti-cancer therapeutic research. Exploring models of various cancer types will help understanding cancer and the corresponding therapeutic approach. It can lead to personalized and efficient treatments.

There are several models developed for different type of tumor evolutions and therapy efficiency. In^4^ are reviewed several emerging therapeutic strategies and discussed how mathematical models have contributed to the design of such schedules. All researcher in the field agree that mathematical models can be used to describe and forecast the behavior of cancer. This is one of the main objectives of “mathematical oncology”^5–7^. The origins of mathematical oncology can be considered the Gompertz population growth model^8^. Another milestone is represented by the Bertalanffy’s organism growth model^9^. More recently several models are developed and discussed. An extensive review of recent results proves the ability of such models to simulate and predict the spatiotemporal development of tumors^10^.

As a starting point for the present research, in^11^ can be found the fitting of differential equation models to tumor volume measurements of patients undergoing chemotherapy or cancer immunotherapy for solid tumors. There are compared six classical models which are widely used in the field: the Exponential, Logistic, Classic Bertalanffy, General Bertalanffy, Classic Gompertz and General Gompertz model. The study concludes that these models could potentially be effective at predicting treatment outcome.

On the other hand, fractional order calculus becomes more and more used in biological, chemical and medical systems^12^. It generalizes the classical, integer order differential calculus to non-integer orders. Many researchers have already proved the superior performance of fractional order models for describing this phenomenon. Great results are presented in different areas: from biosciences, bioengineering, medicine, economics to physics, control, signal processing, neural networks^13–16^. Many authors agree that fractional operators are useful in capturing and understanding the more relative consequences of physical phenomena with higher nonlinearity and complexity with long-range memory and history-based properties^17^. For example paper^18^ analyzes the dynamics of a fractional partial differential equation model of Zika virus. Incorporating the diffusion phenomena using Atangana–Baleanu fractional derivative, the authors explain how the spread of humans and mosquitoes influences the disease's transmission.

On the other hand mathematical oncology is both an old and a new field of research. Recently, multidisciplinary approaches became more and more attractive. Predictive mathematical modeling is widely used to fill in gaps between mathematicians, clinicians, biologist and to make predictions of cancer progression and response to therapy on a patient-specific basis. Many papers are published in the field, but all researchers agree that mathematical oncology is still in the early stage. Any contribution to the field is welcomed.

The present work try to combine this two field by extending the classical tumor growth models using the fractional differential operator. The results are compared by analyzing the mean squared errors (MSE) with both real data and the integer order models, highlighting the advantages introduced by fractional calculus. The obtained results show the utility and ability of fractional order models to emphasize certain characteristics that the integer order systems cannot describe.

The paper is structured as follows. After this introductory part, Section “Materials and methods” presents the used methods, while Section “Results and discussions” discusses the obtained results. The work ends with a concluding section.

## Materials and methods

The concept of integral operator is defined in several ways^19^. The most used is the Riemann–Liouville definition, which states that a fractional order integral of order *ℜ*(*α*) > 0 is a natural consequence of Cauchy’s formula for repeated integrals, expressed as^19^:

1 $$ I^{n} f\left( x \right) = \frac{1}{n!}\mathop \smallint \limits_{a}^{x} f\left( t \right) \cdot \left( {x - t} \right)^{n - 1} {\text{d}}t, $$

where *I* represents the notation for the integral operator and *n* is a natural number, the order of integration. As it can be seen, there is a constraint regarding the order *n* of this operator, due to the term *n!*. Introducing the Gamma function in the above formula, the notion of integration order can be extended from the set of natural numbers to the set of positive real numbers, so the new form of the integral becomes:

2 $${I}^{\alpha }f\left(x\right)=\frac{1}{\Gamma \left(\alpha \right)}{\int }_{a}^{x}f\left(t\right)\cdot {\left(x-t\right)}^{\alpha -1}dt,$$

where $\alpha \in {\mathbb{R}}_{+}$ is the new order of integration and

$$ \Gamma \left( \alpha \right) = \int\limits_{0}^{\infty } {t^{\alpha - 1} e^{ - t} dt} $$

is the Euler’s *Gamma* function which is a generalization of a factorial. The generalization for the whole set of real numbers becomes:

3 $${I}^{\alpha }f\left(x\right)=\frac{1}{\Gamma \left(-\alpha \right)}{\int }_{a}^{x}f\left(t\right)\cdot {\left(x-t\right)}^{-\alpha -1}dt,$$

which describes the real order integral operator, keeping at the same time the inverse correspondence between differentiation and integration.

This definition became^19^:

4 $$ {\text{I}}_{{}}^{\alpha } f(t) = \frac{1}{\Gamma \left( \alpha \right)}\int\limits_{0}^{t} {\left( {t - \tau } \right)^{\alpha - 1} f(\tau )d\tau } ,\;t > 0, \, \alpha \in R^{ + } , $$

for dynamic systems, where *f*(*t*) is a causal function of *t*.

The fractional-order derivative of order *α ∈ *R + can be defined using the Riemann–Liouville formula^19^:

5 $$ {}_{R}{\text{D}}^{\alpha } f(t) = \frac{{d^{m} }}{{dt^{m} }}\left[ {\frac{1}{{\Gamma \left( {m - \alpha } \right)}}\int\limits_{0}^{t} {\frac{f\left( \tau \right)}{{\left( {t - \tau } \right)^{\alpha - m + 1} }}d\tau } } \right],\;m - 1 < \alpha < m,\;m \in N $$

or by the alternative definition introduced by Caputo^16^:

6 $$ {}_{C}{\text{D}}^{\alpha } f(t) = \frac{1}{{\Gamma \left( {m - \alpha } \right)}}\int\limits_{0}^{t} {\frac{{f^{(m)} \left( \tau \right)}}{{\left( {t - \tau } \right)^{\alpha - m + 1} }}d\tau } ,\;m - 1 < \alpha < m,\;m \in N. $$

The advantage of Caputo fractional derivative^20^ is allowing the initial and boundary conditions to be included in the problem. Moreover, this form of the integral overcomes the Riemann–Liouville integral drawback of having the derivative of a constant different from zero.

**Figure 1:**
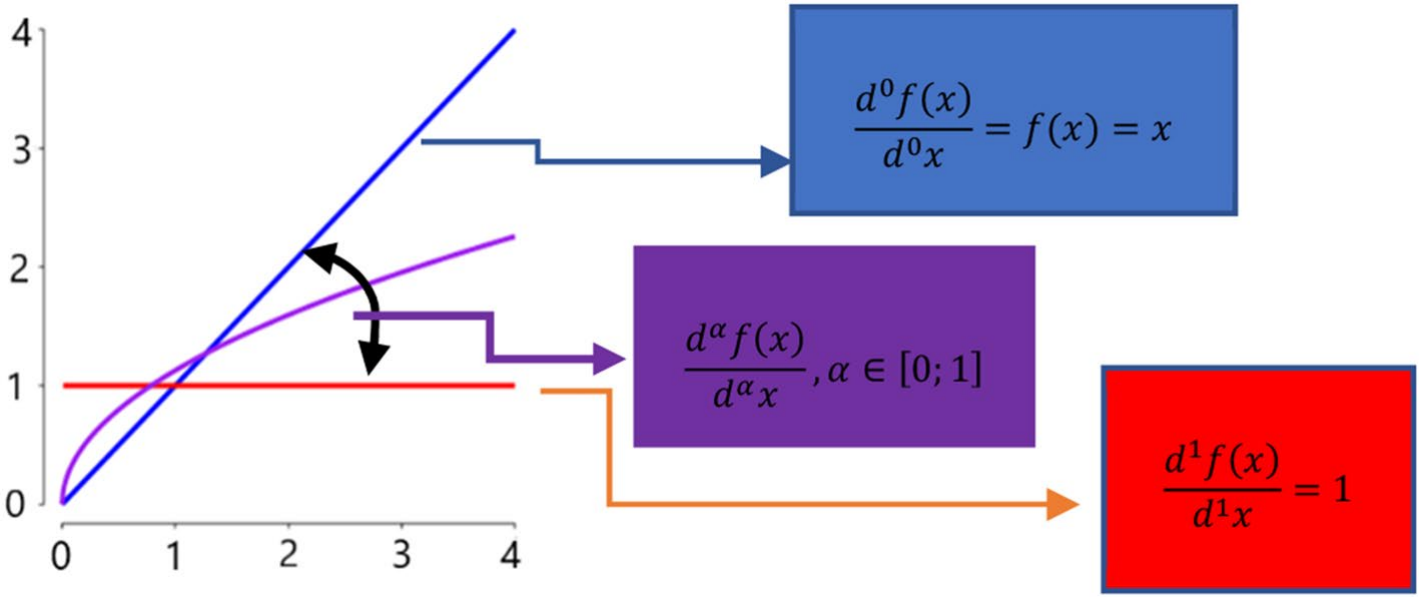
Effect of differentiation order variation.

A useful aspect of the variation of the integration order can be represented intuitively in Fig. 1 where a simple function of the form $f\left(x\right)=x$ is considered. It is obvious that the first order derivative is 1, while the derivative of 0 is the function itself. Varying the integration order between 0 and 1 we can obtain a function that oscillates between the two curves as shown in Fig. 1.

Fractional operators are widely used to describe complex behaviors of dynamical systems^21,22^ and proved to be a powerful tool in describing and understanding the complex behavior of nonlinear systems.

The mathematical models established and discussed in present work are deterministic models. A deterministic model allows to calculate a future event exactly, without the involvement of randomness. This class of models can predict the outcome with certainty which from the beginning may be a wrong assumption regarding the complex behavior of cancer but can provide a good starting point for finding new models and to extend the research to stochastic models. For all models, the dependent variable is the volume of the tumor as a function of time.

The first model considered is the exponential model. This type of model has the simplest form and is based on the following differential equation^6^:

7 $$\frac{dv}{dt}=a\cdot v\left(t\right),$$

where *v* is the tumor volume, $a$ represents the growth exponent, the kinetic parameter.

The second analyzed model is the logistic model, with general form^6^:

8 $$\frac{dv}{dt}=a\cdot v\left(t\right)\cdot \left(1-{\left(\frac{v\left(t\right)}{k}\right)}^{b}\right),$$

where *a* is the inherent growth rate, *k* is the average population size of a species in a habitat (in this case, the volume of cancerous cells in a living organism) and *b* is an exponent that corrects the expression of tumor growth rate.

The third model is the Gompertz model^23^:

9 $$\frac{dv}{dt}=a\cdot v(t)\cdot \mathrm{ln}\left(\frac{b}{v\left(t\right)+c}\right),$$

which is practically a generalization of the logistic model. The constant *c* introduces the volume minimum carrying capacity. Its advantages are proved mainly in the field of biology, helping to describe the evolution of animals and plants, as well as the number or volume of bacteria or cancer cells in a living organism.

The fourth model is the Bertalanffy–Pütter model, having the form^24^:

10 $$\left\{\begin{array}{l}\frac{dv}{dt}=p\cdot {v}^{a}-q\cdot {v}^{b}, for\, a\ne b \\ \frac{dv}{dt}=p{v}^{a}-\mathrm{ln}\left(v\right)\cdot q\cdot {v}^{a}, for\, a=b \end{array}\right.,$$

where the first definition represents the generalized form of the Bertalanffy-Pütter model, the second standing for the particular form when the exponents *a* and *b* are equal. The parameters in this case are the pair of exponents *(a, b)* and coefficients *(p, q)* where *p* is the intrinsic growth (meaning the number of new cells minus the number of dead cells during a generation) and *q* is the growth rate declaration factor of antiangiogenic process (referring to the vascularization process).

However, these models do not include the effects of memory, which are found in biological systems. Hence, in order to take into account the memory effects by the mathematical formulation, it is introduced a new form for each model by replacing the ordinary derivative with the fractional operator. For each model the fractional order differential correspondent is proposed.

The fractional order exponential model:

11 $$ \frac{{d^{\alpha } v}}{{{\text{d}}t}} = a \cdot v\left( t \right), $$

the fractional order logistic model:

12 $$ \frac{{d^{\alpha } v}}{{{\text{d}}t}} = a \cdot v\left( t \right) \cdot \left( {1 - \left( {\frac{v\left( t \right)}{k}} \right)^{b} } \right), $$

the fractional order Gompertz model:

13 $$ \frac{{d^{\alpha } v}}{{{\text{d}}t}} = a \cdot v\left( t \right) \cdot \ln \left( {\frac{b}{v\left( t \right) + c}} \right), $$

and the fractional order Bertalanffy-Putter model, in both generalized and particular form:

14 $$ \left\{ {\begin{array}{l} {\frac{{d^{\alpha } v}}{{{\text{d}}t}} = p \cdot v^{a} - q \cdot v^{b} , for\, a \ne b } \\\\{\frac{{d^{\alpha } v}}{{{\text{d}}t}} = pv^{a} - \ln \left( v \right) \cdot q \cdot v^{a} , for\, a = b } \\ \end{array} } \right.. $$

The existence and unicity of the above-mentioned fractional order models of tumor growth are proved, starting from the following form of Riemann–Liouville integral:

$${I}^{\alpha }={\int }_{x0}^{x}{f\left(t\right)(x-t)}^{\alpha -1}dt, \alpha \in {\mathbb{C}},Re\left\{\alpha \right\}>0$$, respectively

15 $${D}^{-\alpha }f\left(x\right)={\int }_{x0}^{x}{f(t)(x-t)}^{\alpha -1}dt.$$

Because definition (15) makes the derivative of order $\alpha$ of a function $f\left(x\right)=K$, where *K* is constant, to be ${D}^{\alpha }f\left(t\right)={\left[{D}^{\alpha -1}f\left(t\right)\right]}^{^{\prime}}=\frac{K{x}^{\alpha }}{\Gamma \left(1-x\right)}\ne 0$, the following definition has been proposed, by changing the kernel from ${\left(t-\zeta \right)}^{-\alpha }$ to ${e}^{-\frac{t-\alpha }{1-\alpha }}$ and $\frac{1}{\Gamma \left(1-\alpha \right)}$ to $\frac{1}{\sqrt{2\pi \left(1-{\alpha }^{2}\right)}}$, thus obtaining the Caputo-Fabrizio definition of the integral operator^25,26^:

16 $$ D^{\alpha } f\left( t \right) = \frac{{\left( {2 - \alpha } \right)M\left( \alpha \right)}}{{2\left( {1 - \alpha } \right)}}\mathop \smallint \limits_{a}^{t} {\text{ e}}^{{ - \alpha \frac{t - \alpha }{{1 - \alpha }}}} f^{\prime}\left( x \right)dx, $$

where $M\left(\alpha \right)$ is a constant which depends on $\alpha$.

Using Losada-Nieto proposition and considering ${D}^{\alpha }f\left(t\right)=h\left(t\right),$ it is obtained:

$$f\left(t\right)-f\left(0\right)={I}^{\alpha }h\left(t\right)=\frac{2-2\alpha }{M\left(\alpha \right)\left(2-\alpha \right)}h\left(t\right)+\frac{2\alpha }{\left(2-\alpha \right)M\left(\alpha \right)}{\int }_{0}^{t}h\left(x\right)dx,t>0.$$

In the upcoming demonstration fixed-point theory and Picard-Lindelof technique will be used^27^.

In terms of kernel the following equation holds:

$$v\left(t\right)-v\left(0\right)=\frac{2-2\alpha }{M\left(\alpha \right)\left(2-\alpha \right)}K\left(v,t\right)+\frac{2\alpha }{\left(2-\alpha \right)M(\alpha )}{\int }_{0}^{t}K(v,x)dx,t>0,$$

where *K(v,t)* is one of the models mentioned above (e.g. $K(v,t)=av(t)$).

Having the Picard iterations:

$${v}_{n+1}\left(t\right)=\frac{2-2\alpha }{M\left(\alpha \right)\left(2-\alpha \right)}K\left({v}_{n},t\right)+\frac{2\alpha }{\left(2-\alpha \right)M(\alpha )}{\int }_{0}^{t}K\left({v}_{n},t\right)dx,t>0$$

and rewriting the considered system *K*, results:

17 $$\left\{\begin{array}{l}{D}_{t}^{\alpha }v\left(t\right)=F\left(t,v\left(t\right)\right)=F(t,y\left(t\right))\\ v\left(0\right)={v}_{0},0<t<T\end{array}\right.$$

Considering (16) and (17), the equation can be expressed as:

$$v\left(t\right)=v\left(0\right)+\phi \left(\alpha \right)F\left(t,v\left(t\right)\right)+\psi \left(\alpha \right), \mathrm{where\,} \phi \left(\alpha \right)=\frac{2-2\alpha }{M\left(\alpha \right)\left(2-\alpha \right)}, \psi \left(\alpha \right)=\frac{2\alpha }{\left(2-\alpha \right)M(\alpha )}.$$

### Definition

(Lipschitz function)^28^: Let $t\in \left[0,T\right]$ and $f\left(t,x\left(t\right)\right)$ be a function. $f\left(t,x\left(t\right)\right)$ is a Lipschitz function if there is $\uptheta >0$ so that $\Vert f\left(t,{x}_{1}\left(t\right)\right)-f\left(t,{x}_{2}\left(t\right)\right)\Vert \le \theta \Vert {x}_{1}\left(t\right)-{x}_{2}\left(t\right)\Vert , \forall {x}_{1}\left(t\right), {x}_{2}\left(t\right)\in \mathrm{C}\left(\left[0,\mathrm{T}\right],{\mathbb{R}}\right).$

For example, considering the exponential model, it can stated that:

$$ \begin{gathered} F\left( {t,v_{1} \left( t \right)} \right) - F\left( {t,v_{2} \left( t \right)} \right) = K\left( {t,v_{1} \left( t \right)} \right) - K\left( {t,v_{2} \left( t \right)} \right) = av_{1} \left( t \right) - v_{2} \left( t \right) \Rightarrow \hfill \\ F\left( {t,v_{1} \left( t \right)} \right) - F\left( {t,v_{2} \left( t \right)} \right) \le bv_{1} \left( t \right) - v_{2} \left( t \right), \forall b \ge a \epsilon {\mathbb{R}} \Rightarrow \hfill \\ \Rightarrow F\left( {t,v} \right) {\text{is a Lipschitz function}}.{\text{ Moreover}},{\text{ for }}b \in \left[ {0,1} \right], F {\text{is a contraction}} \hfill \\ \end{gathered} $$

### Theorem 1

^29,30^: System (17) has a solution if $\exists$ $c$ so that $\left(\phi \left(\alpha \right)+\psi \left(\alpha \right)\cdot c\right)\theta <1$.

### Theorem 2

^29,30^: The solution is unique if $1-[\phi \left(\alpha \right)+\psi \left(\alpha \right)\cdot t]\cdot \theta >0$.

### Proof

Let be the map $\rho :C\left(\left[0,T\right],{\mathbb{R}}\right)\to C\left(\left[0,T\right],{\mathbb{R}}\right)$ defined by:

$$\rho \left(v\left(t\right)\right)=v\left(0\right)+\phi \left(\alpha \right)\cdot F\left(t,v\left(t\right)\right)+\psi \left(t\right){\int }_{o}^{t}F\left(x,v\left(x\right)\right)dx.$$

It is known that the space $C\left(\left[0,T\right],{\mathbb{R}}\right)$ which has attached the following norm ${\Vert \varphi \Vert }_{c}=\,\mathrm{sup}\left|\varphi \left(t\right)\right|$, $\mathrm{with }t\in \left[0,T\right], \mathrm{is a Banach space}$.

Considering ${v}_{1}\left(0\right)={v}_{2}\left(0\right)$ and knowing that $\rho \left(v\left(t\right)\right)=v(t)$:

$$ \begin{aligned} \rho \left( {v_{1} \left( t \right)} \right) - \rho \left( {v_{2} \left( t \right)} \right)_{C} = & \phi \left( \alpha \right) \left( {F\left( {x,v_{1} \left( x \right)} \right) - F\left( {x,v_{2} \left( x \right)} \right)} \right) \\ & + \psi \left( t \right)\mathop \smallint \limits_{o}^{t} F\left( {x,v_{1} \left( x \right)} \right) - F\left( {x,v_{2} \left( x \right)} \right)dx_{C} \le \phi \left( \alpha \right) F\left( {x,v_{1} \left( x \right)} \right) - F\left( {x,v_{2} \left( x \right)} \right)_{C} \\ & + \psi \left( t \right)\mathop \smallint \limits_{o}^{t} F\left( {x,v_{1} \left( x \right)} \right) - F\left( {x,v_{2} \left( x \right)} \right)_{C} dx \\ \le & \phi \left( \alpha \right) v_{1} \left( x \right) - v_{2} \left( x \right)_{C} + \psi \left( t \right)b\mathop \smallint \limits_{o}^{t} v_{1} \left( x \right) - v_{2} \left( x \right)_{C} dx \\ = & \phi \left( \alpha \right) v_{1} \left( x \right) - v_{2} \left( x \right)_{C} + \psi \left( t \right)tbv_{1} \left( x \right) - v_{2} \left( x \right)_{C} \Rightarrow \\ \Rightarrow & v_{1} \left( x \right) - v_{2} \left( x \right)_{C} \le v_{1} \left( x \right) - v_{2} \left( x \right)_{C} \left( {\phi \left( \alpha \right)b + \psi \left( t \right)bt} \right) \\ \Rightarrow & v_{1} \left( x \right) - v_{2} \left( x \right)_{C} \left( {1 - \phi \left( \alpha \right)b - \psi \left( t \right)bt} \right) \le 0 \\ \end{aligned} $$

A. Existence of the solution:

Let $y\left(t\right)=v\left(t\right)$ for the considered model $\mathrm{F}\left(\mathrm{y},\mathrm{t}\right)=\mathrm{y}$, with $y\left(0\right)$ the model’s initial condition.

Now the Picard iterations are:

$$ \begin{aligned} y_{n + 1} \left( t \right) = & \frac{2 - 2\alpha }{{M\left( \alpha \right)\left( {2 - \alpha } \right)}}F\left( {y_{n} ,t} \right) + \frac{2\alpha }{{\left( {2 - \alpha } \right)M\left( \alpha \right)}}\mathop \smallint \limits_{0}^{t} F\left( {y_{n} ,t} \right)dx \\ \Pi_{n} = & y_{n} - y_{n - 1} = \frac{2 - 2\alpha }{{M\left( \alpha \right)\left( {2 - \alpha } \right)}}\left( {F\left( {y_{n} ,t} \right) - F\left( {y_{n - 1} ,t} \right)} \right) \\ & + \frac{2\alpha }{{\left( {2 - \alpha } \right)M\left( \alpha \right)}}\mathop \smallint \limits_{0}^{t} \left( {F\left( {y_{n} ,t} \right) - F\left( {y_{n - 1} ,t} \right)} \right)dx \\ \Rightarrow & \Pi_{n} = y_{n} - y_{n - 1} = \frac{2 - 2\alpha }{{M\left( \alpha \right)\left( {2 - \alpha } \right)}}\left( {F\left( {y_{n} ,t} \right) - F\left( {y_{n - 1} ,t} \right)} \right) \\ & + \frac{2\alpha }{{\left( {2 - \alpha } \right)M\left( \alpha \right)}}\mathop \smallint \limits_{0}^{t} \left( {F\left( {y_{n} ,t} \right) - F\left( {y_{n - 1} ,t} \right)} \right)dx. \\ \end{aligned} $$

18 $$ \begin{aligned} \Pi_{n} \le & \frac{2 - 2\alpha }{{M\left( \alpha \right)\left( {2 - \alpha } \right)}}F\left( {y_{n} ,t} \right) - F\left( {y_{n - 1} ,t} \right) \\ & + \frac{2\alpha }{{\left( {2 - \alpha } \right)M\left( \alpha \right)}}\mathop \smallint \limits_{0}^{t} F\left( {y_{n} ,t} \right) - F\left( {y_{n - 1} ,t} \right)dx \Rightarrow \\ \Rightarrow & \Pi_{n} \le \frac{2 - 2\alpha }{{M\left( \alpha \right)\left( {2 - \alpha } \right)}}\theta y_{n - 1} \left( t \right) - y_{n - 2} \left( t \right) \\ & + \frac{2\alpha }{{\left( {2 - \alpha } \right)M\left( \alpha \right)}}\mathop \smallint \limits_{0}^{t} y_{n - 1} \left( t \right) - y_{n - 2} \left( t \right)dx \Rightarrow \\ \Rightarrow & \Pi_{n} \le \frac{2 - 2\alpha }{{M\left( \alpha \right)\left( {2 - \alpha } \right)}}\theta \Pi_{n - 1} + \frac{2\alpha }{{\left( {2 - \alpha } \right)M\left( \alpha \right)}}\mathop \smallint \limits_{0}^{t} \Pi_{n - 1} dx. \\ \end{aligned} $$

Repeating the algorithm above we get:

$$\Vert {\Pi }_{n}\Vert \le \Vert y\left(0\right)\Vert {\left[\frac{2-2\alpha }{M\left(\alpha \right)\left(2-\alpha \right)}+\frac{2\alpha }{\left(2-\alpha \right)M\left(\alpha \right)}\right]}^{n}{\theta }^{n}\Rightarrow \mathrm{there\, are\, continuous\, solutions}$$

Let $$y\left( t \right) - y\left( 0 \right) = y_{n} \left( t \right) - B_{n} \left( t \right)$$ with

$$ \begin{aligned} B_{n} \left( t \right) = & \frac{2 - 2\alpha }{{M\left( \alpha \right)\left( {2 - \alpha } \right)}}\left( {F\left( {y,t} \right) - F\left( {y_{n - 1} ,t} \right)} \right) \\ & + \frac{2\alpha }{{\left( {2 - \alpha } \right)M\left( \alpha \right)}}\mathop \smallint \limits_{0}^{t} \left( {F\left( {y,t} \right) - F\left( {y_{n - 1} ,t} \right)} \right)dx \\ \le & \frac{2 - 2\alpha }{{M\left( \alpha \right)\left( {2 - \alpha } \right)}}F\left( {y,t} \right) - F\left( {y_{n - 1} ,t} \right) \\ & + \frac{2\alpha }{{\left( {2 - \alpha } \right)M\left( \alpha \right)}}\mathop \smallint \limits_{0}^{t} F\left( {y,t} \right) - F\left( {y_{n - 1} ,t} \right)dx \\ \le & \frac{2 - 2\alpha }{{M\left( \alpha \right)\left( {2 - \alpha } \right)}}\theta F\left( {y,t} \right) - F\left( {y_{n - 1} ,t} \right) \\ & + \frac{2\alpha }{{\left( {2 - \alpha } \right)M\left( \alpha \right)}}\mathop \smallint \limits_{0}^{t} F\left( {y,t} \right) - F\left( {y_{n - 1} ,t} \right)dx \\ \le & \frac{2 - 2\alpha }{{M\left( \alpha \right)\left( {2 - \alpha } \right)}}\theta \left\| {y - y_{n - 1} } \right\| \\ & + \frac{2\alpha }{{\left( {2 - \alpha } \right)M\left( \alpha \right)}}\theta \left\| {y - y_{n - 1} } \right\|t \ldots \\ \le & \left[ {\frac{2 - 2\alpha }{{M\left( \alpha \right)\left( {2 - \alpha } \right)}} + \frac{2\alpha }{{\left( {2 - \alpha } \right)M\left( \alpha \right)}}t} \right]^{n + 1} \theta^{n + 1} b \Rightarrow \\ \end{aligned} $$

$\Rightarrow B_{n} \left( t \right) \to 0, {\text{when n}} \to 0 \Rightarrow$ the functions (18) are solutions for the considered sistem.

B. Uniqueness of the solution:
  Assuming $1-\left[\phi \left(\alpha \right)+\Psi \left(\alpha \right)\cdot t\right]\theta >0$, meaning we have a unique solution and also knowing that ${\Vert {v}_{1}\left(x\right)-{v}_{2}\left(x\right)\Vert }_{C}\left(1-\phi \left(\alpha \right)b-\psi \left(t\right)bt\right)\le 0,$ we get that ${\Vert {v}_{1}\left(x\right)-{v}_{2}\left(x\right)\Vert }_{C}\le 0,$ but ${\Vert {v}_{1}\left(x\right)-{v}_{2}\left(x\right)\Vert }_{C}=\mathrm{sup}\left( |{v}_{1}\left(x\right)-{v}_{2}\left(x\right)|\right)\ge 0,$ so this means that ${v}_{1}\left(x\right)={v}_{2}\left(x\right)\Rightarrow$ the solution is unique.
C. Stability of solution:
  For simplicity, we will consider the model (11).
  The differential equation $\frac{{d}^{x}v}{dt}=a\cdot v\left(t\right)$, is a fractional differential equation with a fractional order $x$.

To prove the stability of the solution v(t) to this fractional differential equation, we can use the concept of Mittag–Leffler stability, which is a generalization of Lyapunov stability for fractional differential equations.

Specifically, we can show that the solutions of the fractional differential equation converge to a steady-state solution as time approaches infinity if the Mittag–Leffler function satisfies certain conditions.

The Mittag–Leffler function ${E}_{\alpha }(z)$ is defined as:

$${E}_{\alpha }\left(z\right)={\sum }_{k=0}^{\infty }{z}^{k}/\Gamma \left(\alpha *k+1\right),$$

where $\alpha$ is a constant exponent that determines the rate of decay or growth of the function for large values of z.

The general solution to the fractional differential equation $\frac{{d}^{\alpha }v}{dt}=a\cdot v\left(t\right)$, is given by:

$$v\left(t\right)=A\cdot {E}_{\alpha }((a\cdot t)^x),$$

where A is a constant of integration.

To show the Mittag–Leffler stability of this solution, we need to demonstrate that ${E}_{\alpha }\left({\left(a\cdot t\right)}^{x}\right)$ converges to a finite value as time goes to infinity for certain values of $\alpha$ and *a.*

For $0 < x < 1$, the Mittag–Leffler function ${E}_{\alpha }\left(z\right)$ is monotonically increasing and bounded for $0 <\mathrm{ z }<\mathrm{ infinity}$ and all $\alpha >0$. Therefore, for $0<x<1$ and any *a*, ${E}_{\alpha }((a\cdot t)^x)$ converges to a finite value as *t* goes to infinity.

For $\mathrm{x }\ge 1$, the Mittag–Leffler function ${E}_{\alpha }\left(z\right)$ is not necessarily bounded, but it has a Laplace transform that is bounded for $\mathrm{Re}(\mathrm{s}) > \alpha$, which means that ${E}_{\alpha }\left({\left(a\cdot t\right)}^{x}\right)$ decays exponentially for certain values of $\alpha$ and *a*. Specifically, ${E}_{\alpha }\left({\left(a\cdot t\right)}^{x}\right)$ is bounded for $0 <\alpha < x$ and $a\le 0$, which implies that the solution $v(t)$ = $A\cdot {E}_{\alpha }((a\cdot t)^x)$ is Mittag–Leffler stable for these values of *α* and *a*.

Therefore, we can conclude that the solution $v\left(t\right)$ = $A\cdot {E}_{\alpha }\left({\left(a\cdot t\right)}^{x}\right)$ to the fractional differential equation $\frac{{d}^{x}v}{dt}=a\cdot v\left(t\right)$ is Mittag–Leffler stable for certain values of $\alpha$ and *a*, depending on the order *x* of the fractional derivative.

Having these proofs, all the above models can be taken into account as being valid to describe the biological process considered. All parameter used in the models are supposed to be positive constants, being a biological system. All parameters have the same definitions as in the integer order case, appearing the additional degree of freedom, α, the fractional order.

The used database is from research^31,32^, where tumor pieces from a mouse tumor were transplanted orthotopically to syngeneic FVB mice. The tumor’s volume was measured over a period of 18 days. One group was observed without treatment and a second one was treated based on clinician’s protocol. The treatment consisted in drug injection when the tumor volume reached 200 [mm^3^] and at least 10 days have passed since the last treatment. The injected drug dose used was the maximum tolerable dose of 8 [mg/kg]. If the maximum volume threshold of 2000 [mm^3^] was reached, the experiment was stopped. The observations were made regularly, once every two to three days, the system’s identification being performed on the interpolated data set using spline curves. There were used two sets of measurements to study the tumor’s growth: the first case on which a treatment scheme was applied and the second case where only observations were made on the evolution of the tumor’s volume.

For all enumerated models the dependent variable is considered the volume of the tumor as a function of time. The goal is to fit each type of models to the entire time series for each dataset. The statistical endpoint of each experiment is the minimum Mean Square Error (MSE):

19 $$MSE=\frac{1}{n}\sum_{i=1}^{n}{\left({y}_{i}-{\widehat{y}}_{i}\right)}^{2},$$

where *n* represents the number of samples for each model, *y*_*i*_ are the real data values and ${\widehat{y}}_{i}$ the values estimated by the models. For simplicity as initial conditions were used near zero values.

All model fitting procedures were implemented in Matlab^®^. For the fractional order calculus FOMCON toolbox is used^33^. The optimal parameters for the integer order models were obtained using the *fminsearch* function from Matlab^®^. The fractional order models were used with the same parameters as already fitted for the integer order ones, establishing the best fit by only changing the fractional order. In this way it can be highlighted the effect of the introduced fractional order on the tumor model dynamics.

## Results and discussions

**Figure 2:**
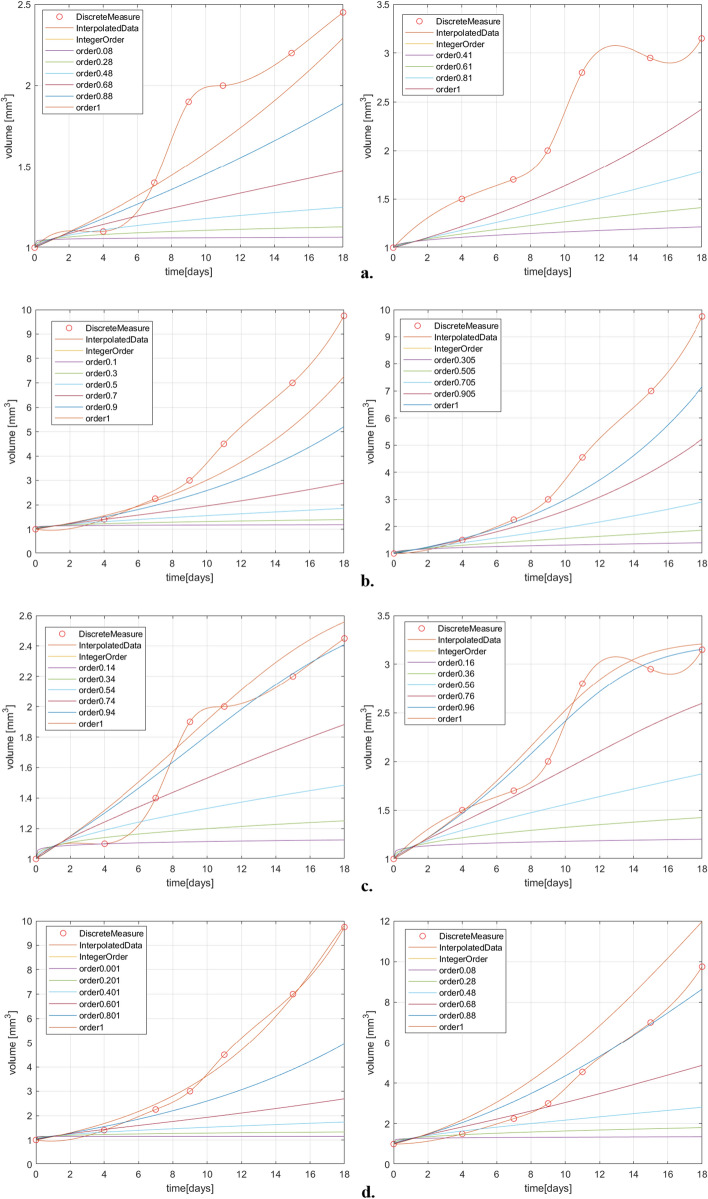

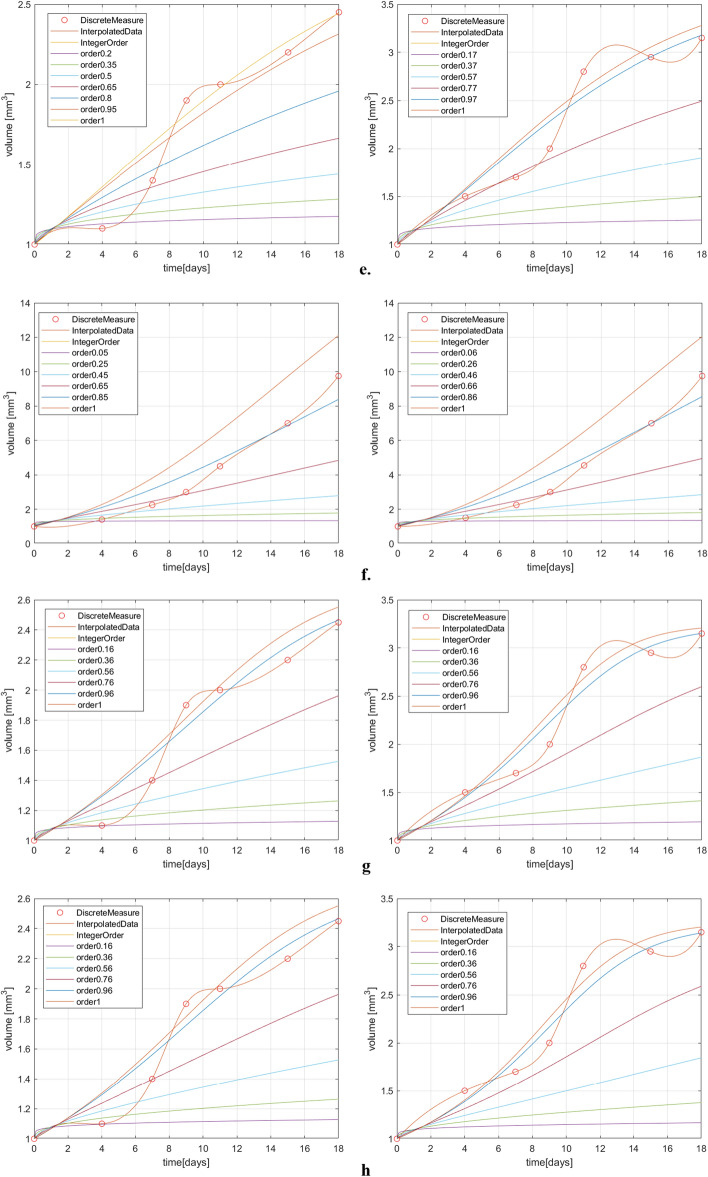

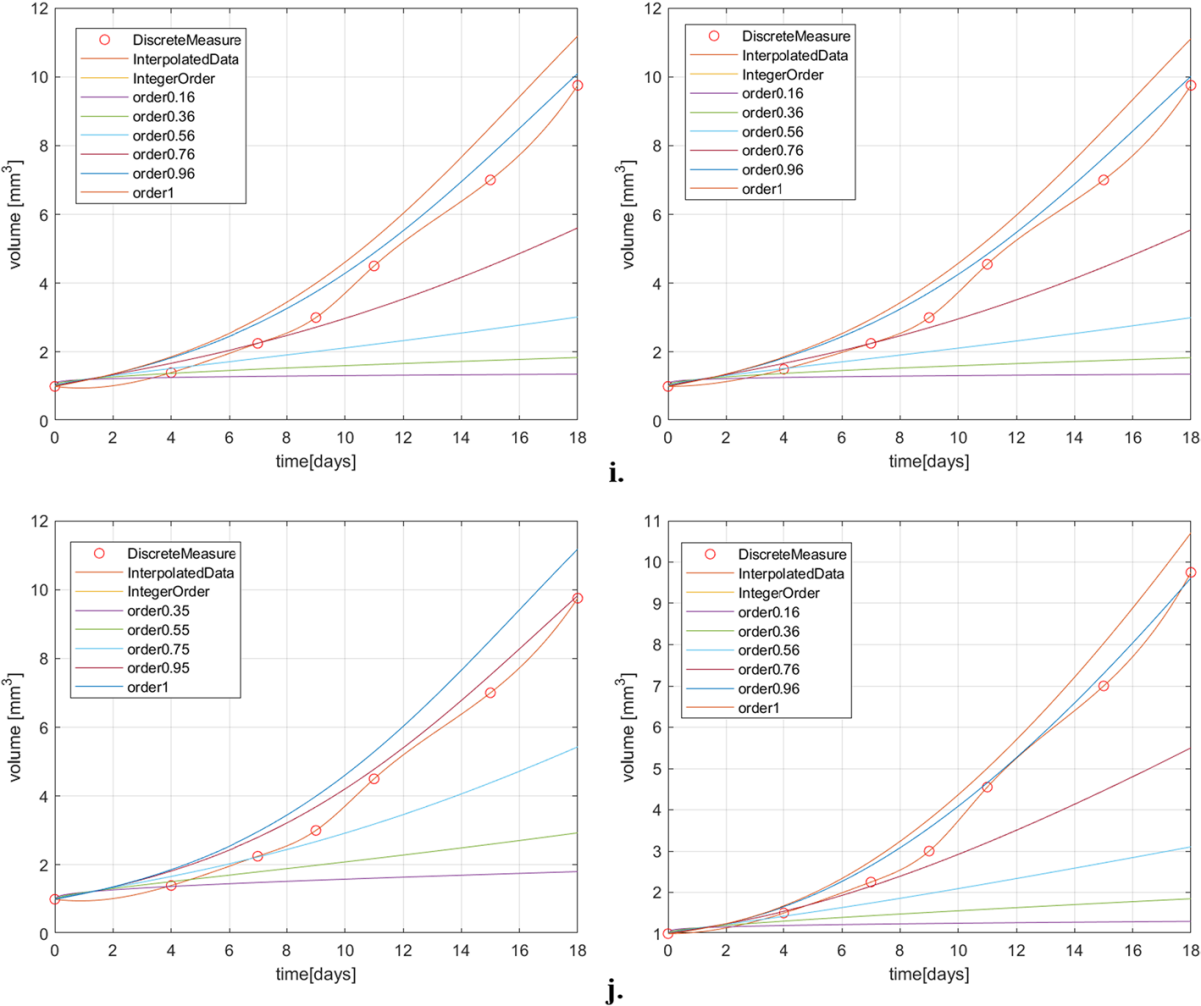
(**a**) Exponential model results for treated case; (**b**) Exponential model results for untreated case; (**c**) Logistic model results for treated case; (**d**) Logistic model results for untreated case; (**e**) Gompertz model results for treated case; (**f**) Gompertz model results for untreated case; (**g**) Generalized Bertalanffy–Pütter model results for the treated case; (**h**) Particularized Bertalanffy–Pütter model results for the treated case; (**i**) Generalized Bertalanffy–Pütter model results for the untreated case; (**j**) Particularized Bertalanffy–Pütter model results for the untreated case.

The simulation results for two dataset for both treated and untreated case are plotted in Fig. 2. These graphics reveal that the fractional order derivative has a significant impact on the dynamics of the tumor evolution.

**Figure 3:**
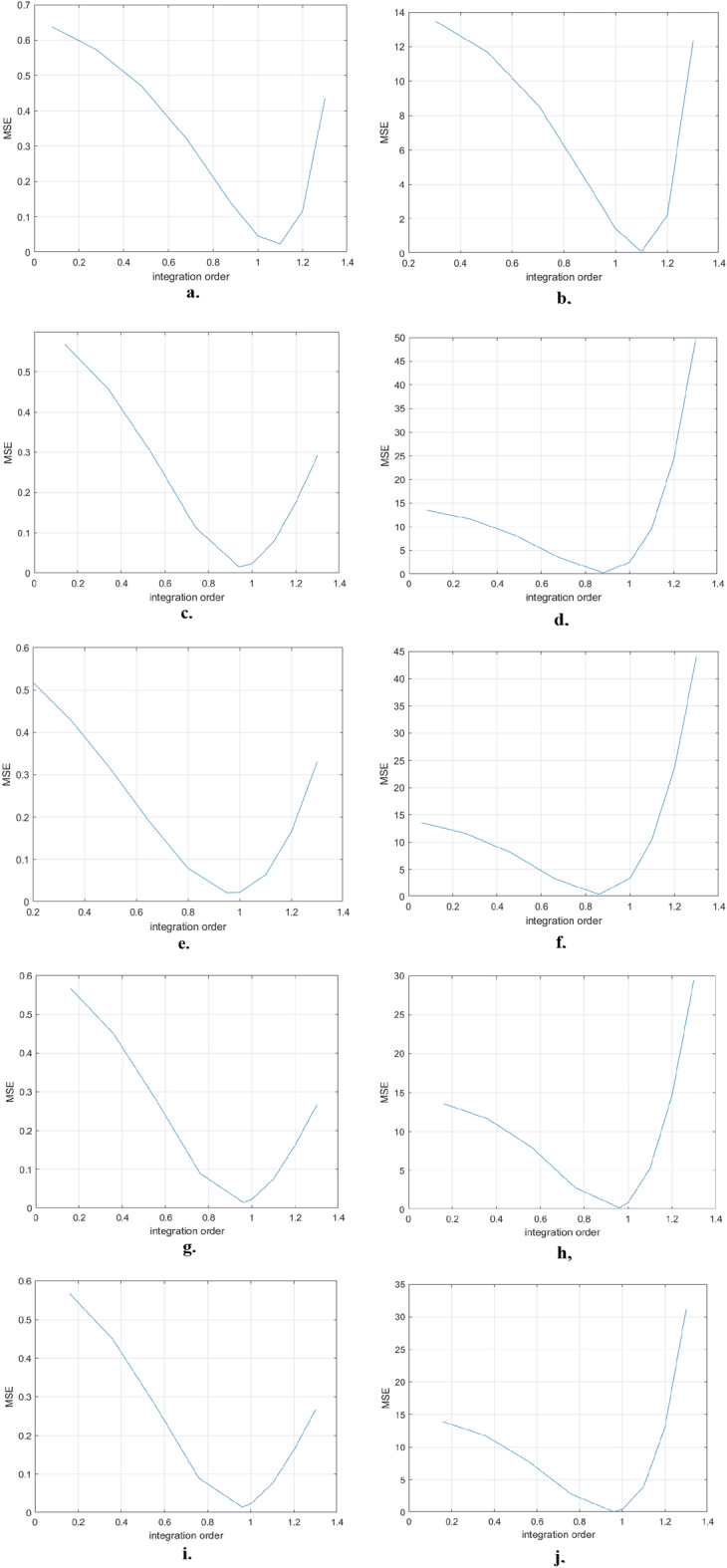
MSE evolution for different fractional orders in the case of the: (**a**) exponential model for treated tumor; (**b**) exponential model for untreated tumor; (**c**) logistic model for treated tumor; (**d**) logistic model for untreated tumor; (**e**) Gompertz model for treated tumor; (**f**) Gompertz model for untreated tumor; (**g**) generalized Bertalanffy–Pütter model for treated tumor; (**h**) generalized Bertalanffy–Pütter model for untreated tumor; (**i**) particularized Bertalanffy-Pütter model for treated tumor; (**j**) particularized Bertalanffy–Pütter model for untreated tumor.

For each considered fractional order, MSE is computed for the resulting models. The impact of the fractional order on the MSE is plotted in Fig. 3. It can be observed that there is an optimum value in each case.

**Figure 4:**
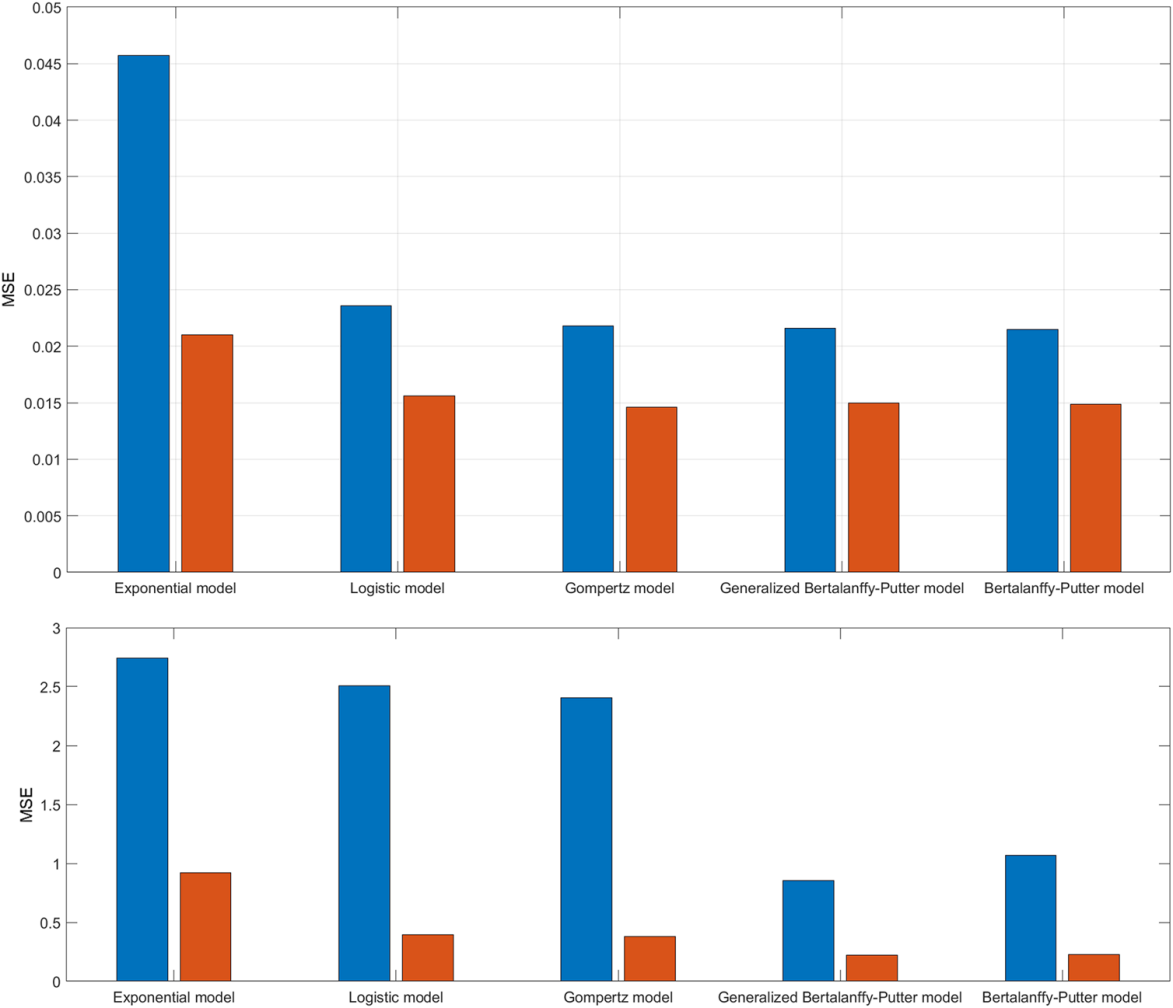
MSE evolutions for the four considered model, both integer and fractional order version: (**a**) the treated data case; (**b**) the untreated data case.

The resulted best fitting integer order models and the corresponding fractional order equivalent, having the same coefficients, are presented in Table 1, while Table 2 highlights the obtained mean squared errors: The evolutions of the MSE for each considered model for both integer and fractional order version for the treated and untreated case data are plotted in Fig. 4. With blue are plotted the integer order model results and with read the fractional order correspondents.

**Table 1:** The obtained models.

	Treated case	Untreated case
Integer order exponential model	$\frac{dv}{dt}=0.046\cdot v\left(t\right)$	$\frac{dv}{dt}=0.109\cdot v\left(t\right)$
Fractional order exponential model	$\frac{{dv}^{0.97}}{dt}=0.046\cdot v\left(t\right)$	$\frac{{dv}^{0.98}}{dt}=0.109\cdot v\left(t\right)$
Integer order logistic model	$\frac{dv}{dt}=0.0735\cdot v\left(t\right)\cdot \left(1-{\left(\frac{v\left(t\right)}{2.81}\right)}^{3.35}\right)$	$\frac{dv\left(t\right)}{dt}=0.565\cdot v\left(t\right)\cdot \left(1-{\left(\frac{v\left(t\right)}{35.2}\right)}^{0.134}\right)$
Fractional order logistic model	$\frac{d{v}^{0.94}\left(t\right)}{dt}=0.0735\cdot v\left(t\right)\cdot \left(1-{\left(\frac{v\left(t\right)}{2.81}\right)}^{3.35}\right)$	$\frac{d{v}^{0.88}\left(t\right)}{dt}=0.565\cdot v\left(t\right)\cdot \left(1-{\left(\frac{v\left(t\right)}{35.2}\right)}^{0.134}\right)$
Integer order Gompertz model	$\frac{dv\left(t\right)}{dt}=0.204\mathit{ln}\left(\frac{8.81}{v\left(t\right)+5.04}\right)$	$\frac{dv\left(t\right)}{dt}=0.106\mathit{ln}\left(\frac{26.09}{v\left(t\right)+2.24}\right)$
Fractional order Gompertz model	$\frac{d{v}^{0.95}\left(t\right)}{dt}=0.204\mathit{ln}\left(\frac{8.81}{v\left(t\right)+5.04}\right)$	$\frac{d{v}^{0.86}\left(t\right)}{dt}=0.106\mathit{ln}\left(\frac{26.09}{v\left(t\right)+2.24}\right)$
Integer order generalized Bertalanffy–Pütter model	$\frac{dv}{dt}=0.168\cdot {v}^{2.18}-0.1\cdot {v}^{2.66}$	$\frac{dv}{dt}=0.894\cdot {v}^{1.38}-0.741\cdot {v}^{1.44}$
Fractional order generalized Bertalanffy–Pütter model	$\frac{d{v}^{0.96}}{dt}=0.168\cdot {v}^{2.18}-0.1\cdot {v}^{2.66}$	$\frac{d{v}^{0.96}}{dt}=0.894\cdot {v}^{1.38}-0.741\cdot {v}^{1.44}$
Integer order particularized Bertalanffy–Pütter model	$\frac{dv}{dt}=0.064{v}^{2.41}-\mathit{ln}\left(v\right)\cdot 0.063\cdot {v}^{2.41}$	$\frac{dv}{dt}= 0.203{v}^{0.74}-\mathit{ln}\left(v\right)\cdot 0.011\cdot {v}^{0.74}$
Fractional order particularized Bertalanffy–Pütter models model	$\frac{d{v}^{0.96}}{dt}=0.064{v}^{2.41}-\mathit{ln}\left(v\right)\cdot 0.063\cdot {v}^{2.41}$	$\frac{d{v}^{0.95}}{dt}= 0.203{v}^{0.74}-\mathit{ln}\left(v\right)\cdot 0.011\cdot {v}^{0.74}$

**Table 2:** MSE for each obtained model.

	Treated case	Untreated case
Integer order exponential model	0.0457	2.74
Fractional order exponential model	0.022	0.94
Integer order logistic model	0.0236	2.506
Fractional order logistic model	0.0156	0.394
Integer order Gompertz model	0.0218	2.4066
Fractional order Gompertz model	0.0146	0.3824
Integer order generalized Bertalanffy–Pütter model	0.0216	0.8557
Fractional order generalized Bertalanffy–Pütter model	0.0150	0.2243
Integer order particularized Bertalanffy–Pütter model	0.0215	1.0682
Fractional order particularized Bertalanffy–Pütter models model	0.0149	0.2273

All obtained results for the integer case are in accordance with the results published in the field^34^. For the fractional order models no results were found in the literature. From the MSE evolution plots for different fractional order values can be concluded that in each case there is an optimum value. Decreasing the fractional order leads to the decrease of MSE until this minimum MSE is reached.

It can be noted that the MSE of fractional order models are reduced at least at half in comparison with the MSE of the integer order equivalent. This good model fitting with fractional order models is even more obvious for the untreated case, where even a 26.21% of MSE reduction can be obtained.

The best integer order model for the treated case is the Gompertz model. However, with the fractional order equivalent a MSE reduction from 0.0218 to 0.0146 is obtained. It is interesting to see, that a MSE not very far from this value (0.021) is obtained with the more simple fractional order logistic model. The extra degree of freedom offered by the fractional order makes a useful tool even from a relatively simple model, like the logistic models.

The best integer order model for the data obtained from untreated tumors is the generalized Bertalanffy-Pütter model, with a MSE of 0.8557. The fractional order equivalent leads to a MSE of 0.2243, which means a 26.21% MSE reduction. As in the untreated case, it can be observed that even the simplest model, the exponential model, can lead to a small MSE if fractional order is used.

After all these tests, performed to answer the question how well classical differential equation models can fit tumor volume trajectories both in treated and untreated case, it is found that in all cases fractional order versions of each model outperforms the corresponding integer order model. Fractional order models are more flexible in fitting empirical data, captures features of compliance data and offer improved model predictions, proved by the reduced MSE.

## Conclusions

Classical mathematical models are in principle useful to model cancer growth for both treated and untreated case. Moreover, although several studies analyze such models on different data, to the best of our knowledge, no fractional order models are developed for tumor models. Deterministic structures can offer a good starting point in finding a proper mathematical model for tumor evolution prediction, but they can be improved by using fractional differential calculus in order to improve the approximation obtained from the integer order differential equation. Fractional calculus is a suitable method in this case due to its memory property, aspect that particularly characterizes biological processes.

The present study discusses the fractional order generalization of four generally recommended mathematical models: exponential, logistic, Gompertz and the generalized and particular form of the Bertalanffy-Pütter model.

As presented in the above results, the best results for the treated tumor case were obtained using the fractional order logistic model, which offers MSE similar to the integer order Gompertz model, recommended in all published studies. This model offers good results for the untreated tumor case as well. For this case the MSE is 0.3002. However, the smallest MSE in this case is offered by the generalized Bertalanffy-Pütter model, having a MSE of 0.2243. It is explicable, being the more complex model, with the most parameters.

Although those models offer a good understanding of the tumor’s growth process and cancer development, they have a major disadvantage by not being able to provide the necessary structure of a model (unlike a state-space model) so that it can be used in developing a control strategy. In addition to this, those models do not include the presence of an input (explicit treatment used on mice), disturbances (changes in the metabolism of the studied specimen or the appearance of certain diseases or abnormalities) or even a resistance to the applied treatment.

To sum up, further research is to be done regarding the use of fractional calculus in control theory, but the preliminary results show their utility and ability to emphasize certain characteristics that the integer order systems cannot observe. Also, stochastic models are proven to be a powerful tool in finding better predictions for processes that are too complex to be described in a deterministic way. Future research include a mix between stochastic and fractional calculus, which can be a solution in finding a proper model to describe any biological process inside a living organism, including cancer.

## Data Availability

The datasets analysed during the current study are available from the corresponding author on reasonable request.
